# Maternal depression symptoms as a context for longitudinal changes in the perinatal brain and infant emotion outcomes

**DOI:** 10.1007/s00737-026-01707-0

**Published:** 2026-05-13

**Authors:** Marybeth McNamee, Sejal Mistry-Patel, Sarah Peoples, Rebecca Brooker

**Affiliations:** 1 Department of Psychological and Brain Sciences, Texas A&M University, College Station, USA; 2 Kinder Institute for Urban Research, Rice University, Houston, USA

**Keywords:** Error-related negativity (ERN), Pregnancy, Maternal depression, Infant emotion, Maternal neuroplasticity

## Abstract

**Purpose:**

Despite theoretical backing, little is known about how contextual factors during pregnancy, which include experiencing increases in symptoms of depression for many women, may affect neural reorganization. We tested within-person stability in a neural process of self-monitoring as a marker of reorganization during pregnancy, whether within-person stability was moderated by maternal depression symptoms, and whether within-person stability had implications for infant postnatal emotion.

**Methods:**

A neural marker of self-monitoring (Error-Related Negativity; ERN) and depression symptoms were assessed in 91 pregnant women. ERN and infant emotion were observed at 4 months postnatal.

**Results:**

Perinatal ERN showed high internal consistency and stability at the group level. However, ERN showed greater stability at high than at low levels of depression symptoms. In addition, when levels of depression symptoms were high, greater prenatal ERN predicted more infant fear.

**Conclusions:**

Results suggest stability in a neural marker of self-monitoring at high levels of depression symptoms, consistent with a possibility that more adverse contexts restrict neuroplasticity. Neural processes sensitive to a context of depression symptoms may have implications for infant emotion.

## Highlights

- The error-related negativity is generally stable across the perinatal period.
- Perinatal depression symptoms moderate stability in maternal error processing.
- Maternal prenatal neural processing interacts with symptoms of depression to predict infant emotion.

Neurobiological changes to the maternal brain begin prenatally and continue through the postnatal period (Hoekzema et al. 2017; Kim et al. 2010). Perinatal patterns of neural reorganization predict mothers’ postnatal mental health (Barba-Müller et al. 2019), though changes in neural structure and function (Servin-Barthet et al. 2025) likely *co-occur* with changes in symptoms beginning during pregnancy (Brooker et al. 2023). This overlap in neurobiological and psychological changes undergirds pregnancy as a sensitive period of maternal development (Bowlby 1973; Rutherford et al. 2018a, b). By definition, sensitive periods are hallmarked by a broad increase in susceptibility to environmental input (Knudsen 2004). As such, prenatal changes in maternal symptoms may comprise a context, not just an outcome, for neurobiological change. Symptoms of depression may be a particularly salient context for maternal neurobiological adaptations, as they are highly prevalent (Bennett et al. 2004; Faisal-Cury et al. 2021) and rise sharply during pregnancy (Astbury et al. 2025; Brooker et al. 2023).

The Stress Acceleration Hypothesis (SAH; Callaghan and Tottenham 2016) suggests that contextual adversity may expedite the maturation of neural systems, conferring short-term advantages, but ultimately resulting in limits on plasticity in an effort to minimize vulnerability to adverse conditions. Over time, accelerated maturation predicts decrements in mental health and emotional functioning (Callaghan and Tottenham 2016). The SAH posits that such changes occur through a pathway involving the hypothalamic-pituitary-adrenal (HPA) axis, which is activated under conditions of stress or distress. Of particular interest, HPA axis dysfunction and heightened distress are also linked to increased symptoms of depression (Menke 2024). In this way, a mother’s experience of her own symptoms of depression may comprise an adverse environment for her own development, with different patterns of changes to the maternal brain visible depending on the level of symptoms that is present. One might reasonably hypothesize less stability (more change) in maternal neural function when symptoms are low and a protracted period of plasticity is maintained.

The significance of changing maternal neurobiology is implicated in structural and functional neuroimaging work. Gray matter volume reductions are observed in the frontal and cingulate cortices during pregnancy (Hoekzema et al. 2022; Pritschet et al. 2024), including the anterior cingulate cortex, the purported neural generator of the Error-Related Negativity (ERN; van Veen and Carter 2002), an event-related potential derived from electroencephalography recordings believed to index self-monitoring (Falkenstein et al. 2000). ERN is relatively stable over time. However, it is unclear if within-person ERN stability (e.g., the tendency for those with high ERN amplitudes to continue to exhibit high ERN amplitudes) may change during sensitive periods like pregnancy. Notably, ERN is modulated by symptoms of depression (Clayson et al. 2020; Moran et al. 2017). If greater levels of depression symptoms are linked to reduced neural plasticity, more symptoms may be linked to greater ERN stability (i.e., less within-person change) during pregnancy relative to low levels of depression symptoms.

Critically, maternal development, including self-regulatory behaviors likely grounded in neural processes indexed by ERN, also has implications for the emotional development of infants. Maternal ERN is associated with parenting behaviors that predict offspring’s own risk for psychopathology (Suor et al. 2022). Thus, it is reasonable to expect depression-related stability or instability in maternal ERN to be linked to facets of offspring early depression risk, including heightened negative emotion (Field 1992; Tronick and Reck 2009).

## Current study

We examined stability (or change) in maternal perinatal ERN. Consistent with a position that ERN is changing during pregnancy, we expected generally high within-person variability in ERN amplitudes during this period, visible as low internal consistency and small correlations among ERN amplitudes over time.

We also tested whether stability in maternal ERN was moderated by maternal depression symptoms during pregnancy. Consistent with tenets of the SAH, we hypothesized that high levels of maternal depression would be associated with greater within-person stability in ERN amplitudes.

Finally, we tested whether interactions between prenatal maternal ERN and depression symptoms interacted to predict postnatal infant emotion outcomes. We hypothesized that when both maternal ERN amplitudes and depression symptoms were high, infants would show high levels of negative emotions.

## Materials and methods

### Participants

Ninety-two pregnant women (henceforth “mothers”) enrolled in a longitudinal examination of peripartum emotion in mothers and offspring (Brooker et al. 2023; Kling et al. 2023). Participants provided informed, written consent for participation. Research was conducted in accordance with the Declaration of Helsinki and approved by the Human Subjects Committee of the [removed for review] Institutional Review Board (#RB011615-FC). Clinical trial number: not applicable. Both primigravida and multigravida women were enrolled. Sample characteristics are reported in the Table 1.

**Table 1:** Sample characteristics

Maternal age	M = 30.49 (SD = 4.22)
Maternal education	
High school graduate	4.3%
Some college	19.5%
College degree	37.0%
Post-baccalaureate education	39.2%
Maternal race	
White	89.0%
Asian	7.7%
American Indian/Alaska Native	1.1%
Black or African American	1.1%
More than one race	1.1%
Maternal ethnicity	
Hispanic or Latino	2.3%
Not Hispanic or Latino	97.7%
Gross annual family income	
<$15,000	8.6%
$15,001 to $20,000	3.7%
$20,001 to $30,000	7.4%
$30,001 to $40,000	11.1%
$41,000 to $50,000	9.9%
$50,001 to $60,000	17.3%
$60,001 to $70,000	6.2%
$70,001 to $80,000	3.7%
$80,001 to $90,000	6.2%
$90,001 or more	25.9%

Mothers reported low numbers of pregnancy and delivery complications (*M* = 3.35, *SD* = 2.22) at the postnatal assessment. Fever (*n* = 7) was the most common prenatal complication, with labor induction (*n* = 21) the most frequent delivery-related risk. Mothers reported using very few medications during pregnancy (*M* = 1.02, *SD* = 1.13) Most infants were full term (*M* = 39.64, *SD* = 1.75 weeks; range 34–44 weeks; 6 preterm infants). Given the low-risk nature of the sample, pregnancy/delivery complications were not considered further.

Mothers completed two prenatal laboratory visits (second trimester: *M* = 21.15 weeks, *SD* = 3.79; third trimester: *M* = 35.92 weeks, *SD* = 1.47) and a postnatal visit at infant age 4 months (*M* = 4.27, *SD* = 0.62). Rolling recruitment resulted in 81 participants in the second trimester, 85 mothers in the third trimester (11 new participants, 6 withdrawals, 1 baby was born early), and 75 mothers at the 4-month postnatal visit (7 additional withdrawals, 3 participants with infants not yet 4 months old when the study was ended because the PI switched institutions).

Procedures were identical across assessment ages. A full list of study measures is available through the Open Science Framework (https://osf.io/dv8pz/). Two weeks before each laboratory assessment, mothers were mailed questionnaires to complete, including an inventory of depression symptoms. At the laboratory, mothers completed a computerized Go/No-go task while neural activity was recorded using electroencephalography (EEG).

## Measures

### Error-related negativity

ERN was derived from EEG recorded at each laboratory visit using a 32-channel BioSemi Active 2 system (Cortech Solutions, LLC; Wilmington, NC) during a Go/No-go task. Data were sampled at 2048 Hz and referenced to the Common Mode Sense and Driven Right Leg electrodes during recording. Horizontal and vertical eye-movements were recorded via flat electrodes at the outer canthi of the left and right eye, and supra and infra orbital sites of the left eye, respectively.

Stimuli were presented in the center of a 23” computer monitor using Presentation^®^ software (Neurobehavioral Systems, Inc.; Berkeley, CA). Participants were instructed to click their mouse quickly when a triangle pointed upward (go stimulus) but to not click anything when a triangle tilted to the left or right (no-go stimuli). Participants completed a practice block of five trials that included feedback about performance. Once practice trials were complete, participants completed seven experimental blocks of 60 trials each for a maximum of 420 trials per participant. Trials were pseudorandomized such that roughly 20% of trials were no-go trials. Each trial was initiated with a 200 ms fixation cross at the center of the monitor, followed by the stimulus presentation for 400 ms.

**Fig. 1:**
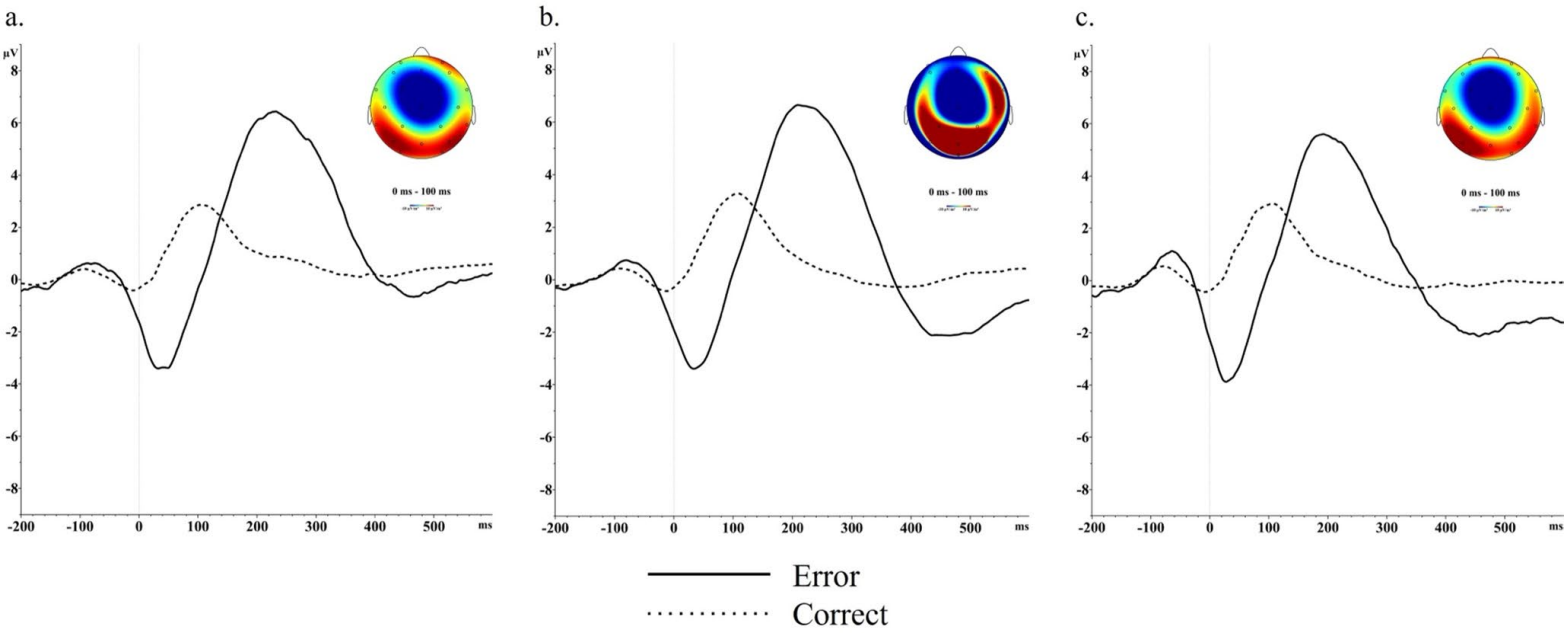
Maternal error-related negativity at electrode Cz across assessments. *Note*. Grand average waveforms for correct and error activity at the second (**a**) and third (**b**) trimesters of pregnancy and four months postnatal (**c**). Topoplots indicate raw error-related negativity (ERN) for the difference in correct and incorrect activity 0–100 ms after a response

EEG data were processed offline using Brain Vision Analyzer (Brain Products GmbH). Following the interpolation of bad channels, continuous EEG was re-referenced to the whole-head average, filtered at 0.1 Hz and 30 Hz, and corrected for eye movements and blinks (Gratton et al. 1983). Error and correct trials were segmented (-200 to 600 ms) and baseline corrected for 200 ms prior to the response. Artifacts were identified and removed using an automated procedure when one of the following criteria were met: a voltage step of more than 75 µV between data points, a difference of 150 µV within 200 ms, amplitudes below 0.5 µV, or activity exceeding + 100µV or -100 µV. For participants with at least 6 trials of usable data (Pontifex et al. 2010), mean area was exported between 0 and 100 ms at electrode Cz based on visual inspection of grand average waveforms and topoplots (Fig. 1). After artifact rejection, mothers had an average of 325 correct *(SD* = 30.0, range = 151–346) and 34 incorrect trials (*SD* = 15.9, range = 7–85) in the second trimester, 322 correct (*SD* = 38.6, range = 138–348) and 39 incorrect trials (*SD* = 25.4, range = 8-148) in the third trimester, and 329 correct (*SD* = 29.7, range = 200–375) and 47 incorrect (*SD* = 25.5, range = 8-146) trials postnatally .

To isolate error-trial activity, residualized ERN (rERN) scores were computed by regressing error trial amplitudes onto correct trial amplitudes and saving the unstandardized residual scores. More negative values reflect a larger rERN while less negative values reflect a smaller rERN.

### Maternal symptoms of depression

Maternal symptoms of depression were assessed at each laboratory visit using the Edinburgh Postnatal Depression Scale (EPDS; Cox et al. 1987), a 10-item measure that asked mothers to rate the degree to which they experienced a variety of symptoms over the last 7 days (e.g., feeling sad) on a 4-point Likert scale (0 = *absence of symptoms*, 3 = *most frequent experience of symptoms*). Internal consistency was acceptable at each visit (second trimester α = 0.82, third trimester α = 0.88, postnatal α = 0.82). The EPDS has demonstrated validity across the perinatal period including pregnancy (Muskens et al. 2022). Mothers’ symptoms ranged nearly the full scale across the three assessments. Symptom counts above the threshold for clinical significance (> 12) were present for 9 mothers (9.8%) in the second trimester, 6 mothers (6.4%) in the third trimester, and 5 mothers (5.3%) postnatally. Maternal depression symptoms were used previously in other work published from this sample (Brooker et al. 2023; Choi et al. 2026; Jennings et al. 2024; Kling et al. 2023).

### Infant negative emotion

Infants’ negative emotion was assessed using a modified Masks episode from the Prelocomotor version of the Laboratory Temperament Assessment Battery (Lab-TAB; Goldsmith and Rothbart 1999). During this episode, infants were presented with four masks in increasing order of putative threat: an evil queen, an old man, a glow-in-the-dark vampire, and a gas mask. Each mask was presented for 10 s with a 5-s pause in between. Mothers were told that they could talk and interact with their baby as they normally would (Crockenberg and Leerkes 2006).

Trained behavioral coders (minimum kappa = 0.70 with master coder R.J.B.) recorded the intensity of facial fear, bodily fear, facial sadness, escape behaviors, and vocal distress across eight 5-s coding epochs. Facial fear and sadness were rated on a 4-point scale (0 = *no codable fear/sadness across three regions of the face* [i.e., mouth, eyes, brows], 3 = *visible fear/sadness across all three regions or an intense expression in two areas*. Vocal distress was rated on a 5-point scale (0 = *absence*, 5 = *maximum intensity cry/scream*). Bodily fear was rated on a 4-point scale (0 = *absence*, 3 = *maximum intensity tensing*). Latency to the first observation of negativity was recorded, in seconds, from the beginning of the episode and then reversed to reflect speed (i.e., higher scores reflected earlier expressions). Ratings across coders were highly correlated (facial fear *r* = .76; facial sadness *r* = .88; vocal distress *r* = .95; bodily fear *r* = .58; latency to fear *r* = .76; latency to sadness *r* = .79) and so were averaged across coders.

Composites were created using regression-based factor scores from a principal components analysis (PCA) containing all coded behaviors. The PCA (orthogonal rotation) returned a two-factor solution accounting for 69.05% of the variance in the original variables (mean communality = 0.69). Each infant received a score reflecting overall fear (facial fear, bodily fear, speed to fear) and one reflecting overall sadness (facial sadness, vocal distress, speed to sadness) with higher scores signifying greater emotional intensity. Scores of infant emotion were used previously in other work published from this sample (Brooker et al. 2020, 2023; Choi et al. 2026; Jennings et al. 2024; Kling et al. 2023).

### Missing data

The most common reason for missing data was nonparticipation at an individual assessment. Of those mothers who participated, ERN data were missing for 18 mothers at the second trimester visit (14 due to technical error resulting in no flags in the data, 1 due to excessive artifacts, 1 due to a recording error that replaced the GNG task with the baseline file, 1 due to too few correct responses, and 1 participant excluded for a deviation from protocol resulting in a child being in the room during the recording). ERN data were missing for 19 mothers at the third trimester visit (8 due to technical error, 10 due to excessive artifacts, 1 due to too few error responses). ERN data were missing for 13 mothers at the postnatal assessment (12 due to technical error, 1 due to a persistent 60 hz noise in the data file). EPDS data were missing from 3 mothers at the second trimester assessment, 3 mothers at the third trimester assessment, and 8 mothers at the postnatal assessment because the mothers either did not complete the questionnaires or skipped items that would artificially result in lower scores. Infant emotion data were missing for 13 infants who did not complete the masks tasks as a result of infants being too young to complete the study before the PI changed institutions or corrupted video data.

Patterns of missing data were consistent with values missing at random (Littles MCAR chi-square = 70.7, *p* = .24). Therefore, missing data for all regression analyses were handled using a full-information maximum likelihood (FIML) procedure (Enders 2010). FIML uses a process of repeatedly estimating likely parameter values across all observed patterns of missing data. Leveraging all available data points, the algorithm identifies the set of parameters that is the best fit to the data as a whole, without needing to impute or replace missing values and allowing one to take advantage of the full sample from which at least some data are available. However, because FIML relies on patterns of available data, scores are not included for participants with missing scores on all variables. One mother did not have data on any of the measures included here; thus, the final analytic sample comprised 91 mothers.

### Plan for analysis

We first tested stability in ERN through three different metrics. First, we examined the internal consistency of ERN amplitudes to understand stability from trial to trial at each assessment. Low internal consistency may indicate a period of change or transition during the trimester under study. Second, we examined test-retest reliability to understand the degree to which ERN amplitudes were stable, across assessments. Low test-retest associations may indicate that a period of change has occurred between assessments. Third, because test-retest reliability may overestimate stability when data are nested (occasion within individual; (Hox 2002), we also calculated an intraclass correlation coefficient for ERN amplitudes across all three assessments.

To test our hypothesis about moderation, we used a multiple regression model to test third-trimester maternal depression symptoms as a moderator of stability between second trimester maternal ERN and postnatal maternal ERN. Finally, we used a path model to test whether third-trimester maternal depression symptoms moderated longitudinal links between second trimester maternal ERN and infants’ negative emotions. Infant sadness and fear were tested as simultaneous outcomes to reduce Type I error; the correlation of infant fear and sadness was set to zero given that they were formed as orthogonal components. Consistent with standard recommendations in the field (Aiken and West 1991), significant interactions were probed at single points (i.e., simple slope; Tabachnick and Fidell 2013) by recentering the continuous variable at values that were + 1 *SD* and − 1 *SD* from the sample mean. This process of recentering allows for a probe of the interaction at different levels of the depression variable, but does not force arbitrary cutoffs or the loss of power that that result from the creation of groups (Aiken and West 1991).

## Results

Preliminary analyses suggested stability in rERN across assessments. Split-half reliabilities were based on the Pearson correlation between the even- and odd-numbered trials for error and correct responses at each assessment and adjusted using the Spearman-Brown prophecy formula to correct for the artificial decrease in reliability that results from splitting the number of trials in half. Internal consistencies in the second trimester (error *r* = .87, correct *r* = .96), third trimester (error *r* = .90, correct *r* = .97), and postnatally (error *r* = .92, correct *r* = .97) were excellent.

Bivariate Pearson correlations suggested that second trimester rERN was significantly associated with third trimester rERN and postnatal rERN (Table 2). Similarly, third trimester rERN was significantly associated with postnatal rERN. Fisher *r-*to-*z* transformations indicated that correlations among rERN were not significantly different across trimesters (*p* > .05), suggesting stability over time. An ICC (ICC = 0.78, 95% *CI* [0.63–0.89]) grounded in our study design nesting assessments within individuals similarly indicated a large degree of variability at the level of the mother (between-individual variance) relative to between assessments (within-individual variance across assessments), indicating good stability in ERN amplitudes over time (Koo and Li 2016).

**Fig. 2:**
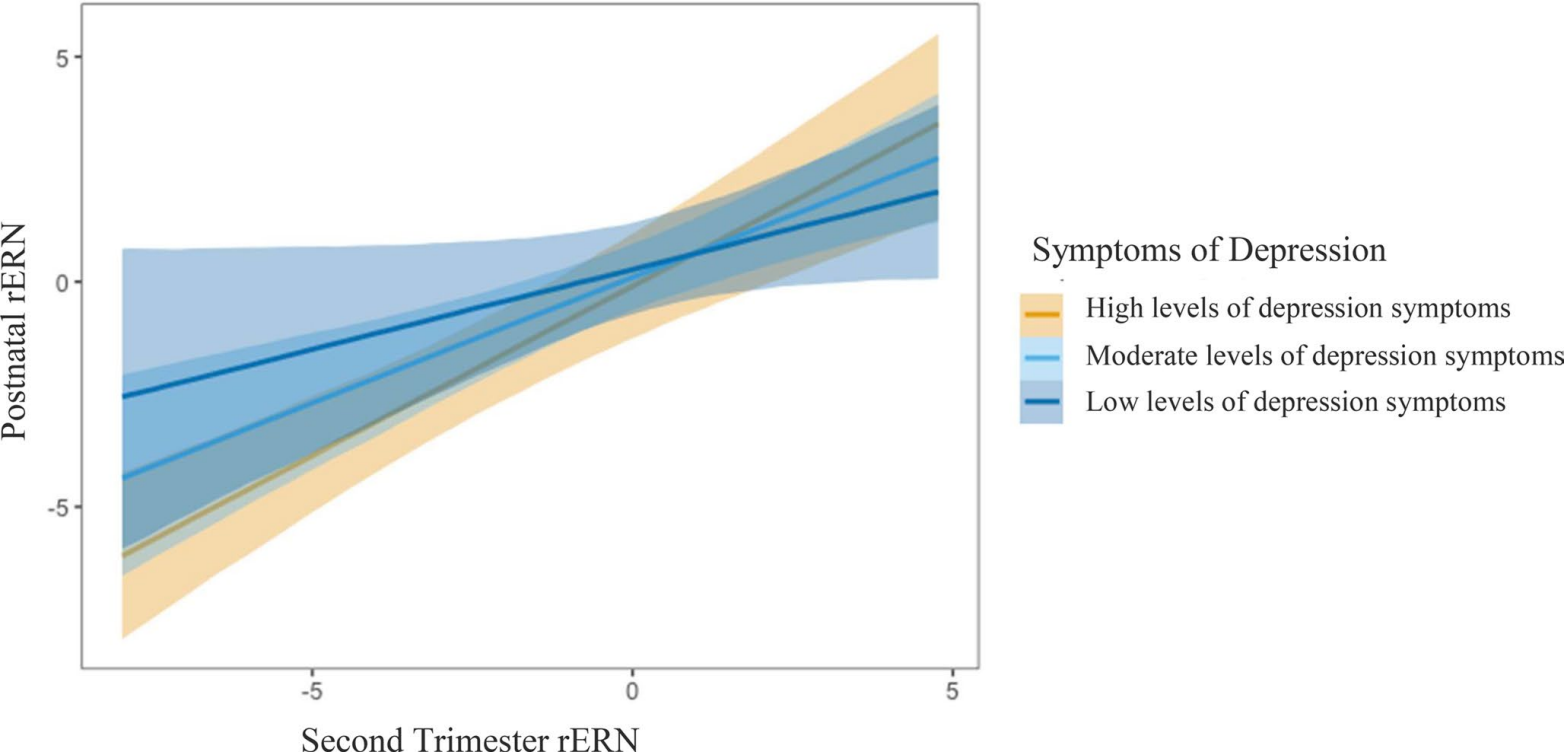
Third trimester depression interacts with prepartum to postnatal error-related negativity

Results from a multiple Ordinary Least Squares (OLS) regression analyses indicated that maternal symptoms of depression interacted with prenatal rERN (β = 0.30, *SE*(β) = 0.12, *p* = .01; Table 3) to predict postnatal rERN. A larger prenatal rERN was linked to a greater postnatal rERN at high (β = 1.54, *SE*(β) = 0.30, *p* < .001) but not low levels of depression symptoms (β = − 0.22, *SE*(β) = 0.40, *p* = .58; Fig. 2).

**Table 2:** Descriptive statistics and bivariate correlations

	*n*	Range	*M*	*SD*	1	2	3	4	5	6	7
1. Second trimester rERN	62	-9.2–4.8	.00	2.96							
2. Third trimester rERN	66	-10.3–5.3	0.00	2.97	0.73^*^						
3. Postnatal rERN	47	-8.9–4.5	0.00	2.86	0.76^*^	0.75^*^					
4. Second trimester depression symptoms	78	1–16	6.42	4.10	− 0.06	0.04	− 0.19				
5. Third trimester depression symptoms	82	0–25	5.46	4.74	− 0.15	0.08	− 0.22	0.48^*^			
6. Postnatal depression symptoms	67	0–21	5.30	4.26	0.08	0.19	− 0.10	0.35^*^	0.32^*^		
7. Infant Sadness	62	-1.7–2.9	0.00	1.00	− 0.01	0.28^+^	0.08	− 0.03	0.09	0.09	
8. Infant Fear	62	-2.6–2.0	0.00	1.00	− 0.08	− 0.13	− 0.15	− 0.18	− 0.02	0.07	0.00

Table reflects observed values without FIML estimation. Infant sadness and fear are standardized variables, rERN values are residuals from a regression in which correct trial ERN amplitudes predict incorrect ERN trial amplitudes.

*p < .05 + *p*<.10

**Table 3:** Maternal depression symptoms moderate ERN stability

	*B*	*SE*(*B*)	95% *CI*
Maternal symptoms of depression	.02	.09	-.16, .20
Second trimester rERN	0.62	0.10	0.42, 0.82
Interaction: Maternal symptoms of depression BY second trimester rERN	0.06	0.02	0.02, 0.11

**Fig. 3:**
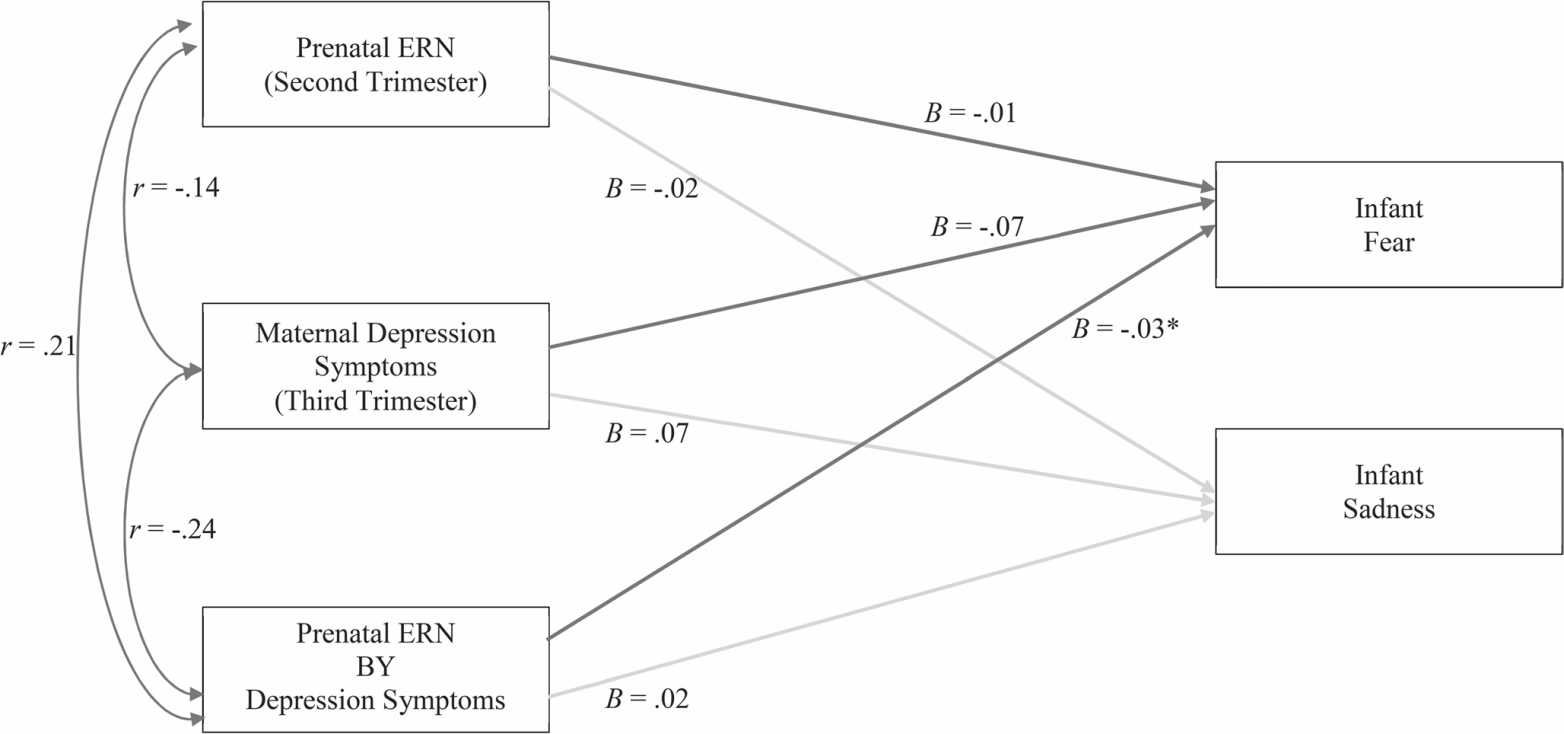
maternal depression as a moderator of links between prenatal ERN and infant emotion. *Note: N* = 92, Path estimates are unstandardized, ^***^p < .05, ^+^*p*<.10

A separate OLS regression revealed a significant interaction between rERN and maternal symptoms of depression predicting infant fear (β = − 0.35, *SE*(β) = 0.15, *p* = .02; Fig. 3). A larger (more negative) ERN was associated with more infant fear when maternal symptoms of depression were high (β = -1.04, *SE*(β) = 0.42, *p* = .01). ERN was unrelated to infant fear when symptoms were low (β = 1.01, *SE*(β) = 0.51, *p* = .06). The interaction between rERN and maternal symptoms of depression predicting infant sadness was not significant (β = 0.30, *SE*(β) = 0.16, *p* = .07).

### Post-hoc analyses

Given our hypothesis about prenatal change and prenatal context, a need to focus on prenatal measures was clear. Given possible questions about the selection of depression symptoms from the third, rather than second, trimester and the use of second, rather than third, trimester ERN, we note that the primary association of interest was longitudinal (prenatal ERN to postnatal ERN). As such, it was important for us to maintain the temporal ordering of the ERN variable. We chose the second trimester ERN in order to maximize longitudinal prediction for potential change. In addition, because we were concerned that including a depression measure concurrent with the ERN assessment could bias results toward the appearance of a longitudinal association when the true interaction was cross-sectional, we chose to maintain the temporal ordering of the variables such that the ERN assessments were at the first and last occasions and the depression measure occurred at an independent prenatal assessment.

In response to reviewer concerns, we reran OLS regression analyses *post hoc* using all possible pairings of the ERN and depression variables. We found that second trimester depression did not significantly moderate the association of second (β = 0.06, *SE*(β) = 0.14, *p* = .66) or third trimester ERN (β = 0.05, *SE*(β) = 0.14, *p* = .73) with postnatal ERN. The interaction between third trimester ERN and third trimester depression predicting postnatal ERN was marginally significant (β = 0.22, *SE*(β) = 0.13, *p* = .09).

## Discussion

Internal consistency estimates and test-retest reliabilities suggested that any neural reorganization that may be occurring is generally not visible at the group level and largely does not affect within-task stability, supporting ERN’s utility as a predictor of individual differences in emotion outcomes (Klawohn et al. 2020). However, maternal symptoms of depression moderated the within-person stability of ERN over time. Specifically, ERN was stable from second trimester to 4 months postnatal only when levels of maternal symptoms were high. This pattern of results is consistent with the notion of pregnancy as a period of plasticity during which neural processes like self-monitoring may undergo change, but highlights that changes may be more protracted in less negative contexts. Consistent with the SAH, plasticity may be temporally limited under less ideal conditions, producing an appearance of greater stability over time.

Because this work was intended to test an initial hypothesis about maternal symptoms as a context for mothers’ development, it is difficult to say whether similar patterns of heightened stability would be expected in the presence of other psychological syndromes across the perinatal period. Indeed, as work continues to illuminate the prevalence of conditions like maternal anxiety (Hunter et al. 2024) and birth-related trauma (Dekel et al. 2017), investigations of other symptoms will be an important extension of our work. Similarly important will be longitudinal studies with additional assessments that can more precisely delineate periods of stability and change within different contexts.

Even so, our findings are consistent with previous MRI studies demonstrating structural changes to the maternal brain across the peripartum period (Hoekzema et al. 2017, 2022; Kim et al. 2010); our findings extend this work in three important ways. First, our findings suggest possible contextual moderators of changes in the maternal brain that may enable more nuanced tracking of changes to mothers’ brains over time. Second, our framework provides a reminder that maternal symptoms can serve as a context for mothers’ own development and emphasize the complex, multifaceted nature of the developmental context. Our focus on the ERN extends the focus of maternal neuroplasticity from structural changes to putative alterations in specific neural processes. In this way, our findings complement work tracking pregnancy-related chances in neural markers of infant face processing (Maupin et al. 2015; Rutherford et al. 2018b), extending a literature that has largely focused on event-related potentials linked to maternal sensitivity (Bernard et al. 2018) and mother infant bonding (Dudek et al. 2018) to also include maternal symptoms of depression. Given that these elements (maternal-infant bonds, maternal sensitivity, maternal depression) are not entirely independent (Campbell et al. 2004), there will be a benefit to future efforts to understand co-developing, interactive, and contingent changes in the maternal brain.

It is also noteworthy that maternal symptoms of depression and prenatal ERN interacted to predict infant emotion outcomes. Effects that included second trimester ERN parallel other work highlighting unique links between maternal depression in the third trimester and infant negative emotion (Davis et al. 2007). Our work expands previous efforts by highlighting a conditional process and putative neural pathway in parent-to-child effects. Specifically, at high levels of maternal depression, a greater ERN was associated more infant fear. A link between heightened ERN and infant fear connects an established neural phenotype of anxiety in adults (Meyer 2016; Moser 2017) with a validated risk factor for anxiety problems in infancy (Brooker et al. 2013, 2016; Buss 2011). Though null effects are notoriously difficult to interpret, a nonreplication of a similar interactive effect predicting infant sadness may suggest that the combination of ERN and maternal symptoms do not predict all infant negative emotion outcomes in equivalent ways.

An overall pattern of effects including post-hoc analyses indicates possible a specificity of results to third trimester depression. Though this is the first study, to our knowledge, to assess the role of third trimester depression symptoms in maternal neurodevelopment, previous empirical work indicates some specificity of third-trimester depression on infant outcomes (Davis et al. 2004; Luoma et al. 2001, 2004), particularly girls (De Bruijn et al. 2009). One possible reason for some level of specificity to third trimester effects may be that elevated estrogen levels interfere with the breakdown of maternal cortisol, further elevating cortisol levels that are escalating across the normative course of pregnancy. By happenstance, the third trimester assessment occurred, on average, just after the typical peak in estrogen concentration near 32 weeks (*M* = 35.92 weeks). Given that the hypothalamic pituitary adrenal (HPA) axis, the end product of which is cortisol, is a theorized pathway for our hypothesized effects, patterns of prior work are thus aligned with a possibility that effects may be most readily apparent in association with third trimester symptoms.

That being said, our modest sample size and lack of a priori hypotheses about timing differences leave us to consider such a possibility with extreme caution. It is also possible that our finding reflects a statistical artifact related to the broader range and variance of depression symptoms in the third trimester, allowing for more overlap with variance in other variables. Additional work will be needed to add clarity and confidence to any interpretation.

Though this study had many strengths, including a multimethod longitudinal design, moderate sample size, and socioeconomic diversity, it is not without limitations. Our community sample was relatively low risk and racially and ethnically homogenous. Although we did see a full range of symptoms, estimates at the highest levels of depression may be less stable because of fewer data points. In addition, the absence of parity data leaves us unable to determine if significant effects may be driven by primiparous or nulliparous mothers. Nonetheless, our results provide a foundation for additional explorations of mothers’ symptoms of depression as a context for their own development and their infants’ outcomes across the perinatal period.

## Data Availability

The data and syntax that support the results reported in this manuscript are available along with a full list of study measures through the Open Science Framework (https://osf.io/dv8pz/).
